# Influencing factors of depressive symptoms during pregnancy in Beijing, China

**DOI:** 10.3389/fpsyt.2025.1500034

**Published:** 2025-02-14

**Authors:** Xiayue He, Xiaoqian Wang, Guoguang Li, Simin Zhu, Yifan Wu, Xiaotong Sun, Yuze Wu, Bo Hu, Jianyin Wu, Lina Feng, Guihong Li, Lei Zhang, Ning Wang, Xuehan Li

**Affiliations:** ^1^ Department of Psychiatry and Mental Health, Beijing HuiLongGuan Hospital, Beijing, China; ^2^ Department of Obstetrics and Gynecology, Beijing Tsinghua Changgung Hospital, School of Clinical Medicine, Tsinghua University, Beijing, China; ^3^ Department of Psychiatry and Mental Health, Fourth People’s Hospital of Liaocheng, Liaocheng, Shandong, China; ^4^ Department of Psychiatry and Mental Health, Yanqing District Mental Health Center of Beijing, Beijing, China; ^5^ Department of Psychiatry and Mental Health, Beijing Daxing Xinkang Hospital, Beijing, China

**Keywords:** pregnancy, influencing factors, perinatal, screening, prenatal depression

## Abstract

**Objective:**

Prenatal depression is a prevalent mental health challenge encountered during pregnancy and is notably associated with adverse maternal and neonatal outcomes. This study aimed to elucidate the likelihood and determinants of depressive symptoms among pregnant individuals in Beijing, thereby laying a foundational framework for the theoretical underpinning of prenatal screening and preemptive interventions for mental disorders.

**Methods:**

The study dataset was derived from 4,564 pregnant individuals in Beijing utilizing a cross-sectional survey methodology. Data collection focused primarily on the personal and obstetric information of the participants. The Edinburgh Postnatal Depression Scale (EPDS) was employed as the primary screening tool to identify depressive symptoms.

**Results:**

The prevalence of depressive symptoms among the included pregnant individuals was 4.1%. The univariate analysis results revealed statistically significant differences in the incidence of depressive symptoms in women with different parities, numbers of births, medication use, numbers of abortions, prepregnancy weights, and body mass indices (BMIs) (the χ^2^ values were 61.130, 52.008, 23.291, 5.293, and 12.681, respectively; P<0.05). There were statistically significant differences in the incidence of depressive symptoms among women with different occupation types (χ^2^ = 30.263, P<0.01). The multivariate logistic regression analysis results revealed that a greater number of pregnancies, number of births, and BMI were risk factors for prenatal depression, whereas the commercial and service worker occupation types were protective factors against prenatal depression.

**Conclusion:**

Future directives should emphasize the enhancement of screening for depressive symptoms among pregnant individuals with greater parity and BMIs, alongside encouraging continued employment and flexible job selection. It is imperative to implement suitable intervention strategies for pregnant individuals exhibiting depressive symptoms to mitigate the incidence of adverse maternal and neonatal outcomes.

## 1 Introduction

Depression, widely recognized as a prevalent, severe, and complex mental health disorder, ranks among the most common mental health challenges globally, affecting more than 300 million individuals (1). The World Health Organization predicted that by 2020, depression would be the second leading cause of global disease burden, with an estimated 10% to 40% of women worldwide experiencing depressive episodes during pregnancy (2). The issue of prenatal depression has increasingly captured international attention, especially given its reported prevalence of approximately 20% in certain developing nations, such as Brazil (3, 4). Data indicate that between 10% and 20% of women exhibit symptoms of depression during and after pregnancy (5). Furthermore, research has highlighted that the incidence of depression ranges from 7.4% to 12.8% across various stages of pregnancy (6), underscoring its emergence as a significant public health concern.

Prenatal depression, a nonpsychotic mental condition characterized by mild to moderate depressive episodes from pregnancy onset through the postpartum period, has clinical symptoms akin to those of depressive disorders. These symptoms include pervasive sadness, anhedonia, and diminished energy, which may or may not cooccur with alterations in sleep and appetite, irritability, diminished sexual interest, psychomotor retardation, and suicidal ideation. However, the clinical presentation of prenatal depression in some women may deviate from the typical presentation, with initial symptoms often including fatigue, sleep disturbances, and appetite changes (4). Women with these atypical presentations are prone to being misdiagnosed with neurasthenia or similar conditions in clinical settings, thereby postponing optimal treatment opportunities and exacerbating the severity of the disorder (4).

Prenatal depression, distinguished as the predominant mental and psychological ailment during pregnancy, not only imposes a significant psychological burden on expectant mothers but may also detrimentally influences the birth process and the health outcomes of fetuses and newborns (7). The adverse effects include a spectrum of complications, such as preeclampsia, premature birth, maternal postpartum depression, and low birth weight, atypical Apgar scores, reduced head circumference, and inhibited growth during the first year of life for infants (8–11). Emerging studies underscore that prenatal anxiety and depression potentially alter brain structure and functionality in infants and children (12). Furthermore, evidence suggests that the offspring of women who experience depression or multiple affective disorders during pregnancy are at heightened risk for the development of behavioral and emotional difficulties, attention-deficit/hyperactivity disorder (ADHD), and autism spectrum disorders in childhood (13–15). These conditions may also increase the risk of mood disorders in adolescence and schizophrenia in adulthood (16, 17). Prenatal depression may have an extensive impact on offspring’s long term trajectories.

During pregnancy, women undergo significant physical and psychological transformations, ranging from the joy of embracing motherhood to career shifts, daily inconveniences, and, for some, the decision to forego their careers to provide childcare. These challenges often precipitate emotional conflicts. In Western countries, extensive research has been conducted on the determinants of prenatal depression, with findings suggesting that racial factors contribute to its prevalence. Specifically, studies indicate a higher incidence of depression among pregnant individuals belonging to ethnic minorities in the Middle East and South Asia (18). Furthermore, factors influencing prenatal depression can be categorized into biological, psychological, and social domains, with body mass index (BMI), family history, pregnancy-related stress, familial support, and personal income identified as potential risk factors (19). However, reports on the consistency of the relationships of these factors with prenatal depression differ (20, 21). A meta-analysis by Dachew BA et al. revealed that there is no significant correlation between total gestational weight gain and symptoms of prenatal depression (22).

Recent research has predominantly focused on sociodemographic and obstetric variables as indicators of prenatal depression. However, many surveys fall short in comprehensively examining sociodemographic and obstetric factors. For example, in most studies, occupational factors are simply classified as employed versus unemployed, with scant statistical analysis of obstetric variables. Moreover, Beijing’s considerable population mobility and the presence of a significant number of nonlocal residents underscore potential regional disparities in the factors influencing prenatal depression. To date, a comprehensive study examining these determinants within this specific geographical context is lacking, highlighting a gap in the understanding of the multifaceted etiology of prenatal depression.

Given the potential profound impact of prenatal depression on expectant mothers and their offspring, this study was designed to assess the prevalence of depression among pregnant individuals in Beijing and to investigate the factors associated with prenatal depression. This effort aimed to provide a theoretical foundation for enhancing the mental health of pregnant individuals. By facilitating more effective screening processes, early identification of mental health anomalies in pregnant individuals can be achieved, enabling primary prevention measures. Such interventions are intended to improve the mental health of pregnant individuals, thereby fostering improved prenatal and postnatal care. This approach underscores the importance of holistic health strategies in supporting both maternal and neonatal well-being.

## 2 Materials and methods

### 2.1 Study design and participants

This cross-sectional study was conducted in Beijing Tsinghua Changgung Hospital from January 2022 to January 2023. Psychiatrists and other doctors at Beijing HuiLongGuan Hospital and Beijing Tsinghua Changgung Hospital conducted psychiatric evaluations and collected basic information. A total of 4,600 pregnant individuals were included and were invited to sign the informed consent form to participate this study. The pregnant individuals completed an on-the-spot questionnaire survey. A total of 4,600 questionnaires were distributed, and 4,600 were recovered, including 36 invalid questionnaires and 4,564 valid questionnaires. The inclusion criteria were as follows: (1) aged 19–48 years; (2) married and a singleton pregnancy(the sample was limited to married participants to avoid scrutiny of unmarried individuals who may experience stigma); (3) no history of mental illness(the sample was limited to individuals without documented history of mental illness, which was ascertained using chart review); (4) no communication barriers and the ability to read and write independently and understand the contents of the questionnaire; and (5) a signed informed consent form. The exclusion criteria were as follows: (1) had a clear mental disorder(as assessed by chart review examining for history of psychiatric disorder); (2) intellectual disability or other impairments preventing understanding of the questionnaire contents; (3) the presence of serious systemic diseases, such as systemic lupus erythematosus, diabetes, cardiovascular and cerebrovascular diseases, liver and kidney diseases, hyperthyroidism, and immunodeficiency diseases(as assessed by chart review); (4) refusal to participate or the discontinuation of participation in the study.

### 2.2 Ethics statement

Ethical review and approval was not required for the study on human participants in accordance with the local legislation and institutional requirements.

### 2.3 Consent statement

Written informed consent to participate in this study was provided by the participants.

### 2.4 Assessment tools and procedures

First, it was essential to collect comprehensive sociodemographic information pertaining to the pregnant participants, which encompassed both personal and obstetric data. The personal data included the age, education level, occupation type, prepregnancy weight, and BMI of the pregnant individuals. The obstetric data included the number of pregnancies, parity (the number of times a woman has given birth), history of spontaneous abortion, occurrences of medical abortion and induced abortion, and any relevant family medical history. This detailed collection of data served as a critical foundation for understanding the participants’ demographic and health-related backgrounds, facilitating a nuanced analysis of the factors associated with prenatal depression.

The Edinburgh Postnatal Depression Scale (EPDS) was utilized to screen for depressive symptoms among the included pregnant individuals. Originally developed by Cox J in 1987, the EPDS was intended for postpartum depression screening, diagnosis, and assessment. Subsequently, the application of the EPDS was extended to evaluate depression symptoms during pregnancy (23, 24), and its effectiveness for pregnant, postpartum, or nonpostpartum individuals has been extensively validated (25, 26). In cohort studies, the Cronbach’s alpha coefficient for EPDS was reported as 0.82, indicating high internal consistency. The EPDS also demonstrated strong test-retest reliability. For instance, retesting the same population within a one-week interval yielded correlation coefficients exceeding 0.8. The design of the EPDS is based on core symptoms associated with postpartum depression, and its content is considered to directly reflect key characteristics of postpartum depression, supporting its strong face validity (27, 28). Owing to its high sensitivity and specificity across various investigations, the EPDS provides a preliminary theoretical framework for screening prenatal depression and has become the predominant screening instrument for epidemiological research of perinatal and postpartum depression (29).

The EPDS is a self-assessment tool comprising ten items that evaluate a range of symptoms, including pessimism, anhedonia, self-reproach, anxiety, fearfulness, impaired functionality, sleep disturbances, sadness, the tendency to cry, and thoughts of self-harm or suicide. Respondents rate the frequency of these symptoms on the basis of their experiences. The scores for the scale are stratified into four levels (0-3 points), with assigned values of 0 (never), 1 (sometimes), 2 (often), and 3 (always). The primary statistical measure is the aggregate score of the ten individual responses, with higher totals indicating greater symptom frequency and more severe depressive symptoms. The evaluation period typically covers the week preceding the consultation. Consistent with the standards established in numerous studies, an EPDS score ≥15 points during pregnancy or ≥13 points postdelivery indicates a high risk for depression (30).

The research team dedicated five days each week to assess the eligibility of pregnant individuals during their scheduled prenatal clinic appointments. For women visiting Chang Gung Hospital in Tsinghua, Beijing, the researchers provided comprehensive information regarding the objectives, methodology, and potential benefits of participating in the study. Pregnant individuals were then informed about their rights and the voluntary nature of their participation. Upon obtaining informed consent from the participants, who willingly signed the informed consent forms, sociodemographic data were collected and the EPDS was administered. It is worth noting that respondents were answering about the past week in this study.

### 2.5 Statistical analysis

EpiData 3.1 software facilitated the scoring of the EPDS and the entry of questionnaire data. Following a double entry and validation process to ensure data accuracy, the information was imported into SPSS version 24.0 for comprehensive statistical analysis. The chi-square test was applied to examine the relationships between the independent variables and the dependent variable of depression. On the basis of the results of the univariate analysis, multivariate unconditional logistic regression analysis was employed to identify factors influencing prenatal depression. A p value less than 0.05 was considered statistically significant. In this analytical context, an odds ratio (OR) greater than 1 indicated that the exposure factor served as a risk factor for prenatal depression (positive correlation), whereas an OR less than 1 suggested that the exposure factor acted as a protective factor against the condition (negative correlation). This methodological approach enabled a nuanced exploration of the variables associated with prenatal depression.

## 3 Results

### 3.1 General information

A total of 4,600 participants participated in this investigation and completed the survey, 4,564 (99.22%) of whom submitted valid responses. The remaining participants were excluded because of incomplete answers or withdrawal from the study. The mean age of the respondents was 34 years (SD: 3.64 years), and the average gestational week was 22.14 weeks (SD: 2.52 weeks). The cohort included 2,756 primiparous women, accounting for 60.39% of the participants, and 1,808 multiparous women, accounting for 39.61%. Among these participants, 187 (4.1%) had scores indicating the presence of depressive symptoms, with an average EPDS score of 6.40 points (SD: 3.85).

### 3.2 Univariate analysis

Univariate analysis included variables such as age, education level, occupation type, number of pregnancies, parity, number of spontaneous abortions, number of medical abortion and induced abortion, family history, prepregnancy weight, and BMI. The analysis revealed that pregnant individuals employed in occupations outside the business and service sectors presented the highest prevalence of depressive symptoms (at 6.38%), whereas those engaged in occupations in the business and service sectors presented the lowest prevalence (at 2.69%), with this difference reaching statistical significance (χ² = 30.263, P<0.01). With respect to the number of pregnancies, a notable increase in depressive symptoms was observed among women with three or more pregnancies, peaking at 7.97%, and this difference was statistically significant (χ² = 61.130, P<0.01). Pregnant individuals with one or more births demonstrated a significantly greater incidence of depressive symptoms (7.19%) than did those with no births (2.70%) (χ² = 52.008, P<0.01). A history of one or more medical abortion and induced abortion correlated with an increased incidence of depressive symptoms (6.46%), which was significantly greater in women with such a history than in women without such a history (3.28%, χ² = 23.291, P<0.01). Pregnant individuals with a prepregnancy weight of ≥56 kg had a depressive symptom incidence of 4.69%, which was significantly greater than the 3.46% reported in those with a prepregnancy weight of less than 56 kg (χ² = 5.293, P=0.021). Similarly, a BMI of ≥21.30 kg/m^2^ was associated with a greater incidence of depressive symptoms (5.08%) than a BMI <21.30 kg/m^2^ (3.11%), and the difference was statistically significant (χ² = 12.681, P<0.01). No significant differences in age, education level, the number of spontaneous abortions, or family history were found between pregnant individuals with and without depressive symptoms, indicating that these factors do not significantly impact the incidence of prenatal depressive symptoms (**Table 1**).

**Table 1:** Characteristics of women with no depressive symptoms and those with depressive symptoms as a function of sociodemographic and obstetric variables.

Variables	N (%)	No depressive symptoms	Depressive symptoms	χ^2^	P
	4564 (100%)	4377 (95.9%)	187 (4.1%)		
Age (years)				0.269	0.874
<34	2271 (49.8%)	2181 (96.04%)	90 (3.96%)		
≥34	2293 (50.2%)	2196 (95.77%)	97 (4.23%)		
Education level				0.245	0.620
High school or below	269 (5.9%)	258 (95.91%)	11 (4.09%)		
Junior college	1000 (21.9%)	962 (96.20%)	38 (3.80%)		
Bachelor’s degree	2288 (50.1%)	2189 (95.67%)	99 (4.33%)		
Master’s degree or above	1007 (22.1%)	968 (96.13%)	39 (3.87%)		
Occupation type^*^				30.263	0.000
Other	439 (9.6%)	411 (93.62%)	28 (6.38%)		
Not working	210 (4.6%)	203 (96.67%)	7 (3.33%)		
State organizations, party-mass organizations, responsible persons of enterprises and institutions	583 (12.8%)	557 (95.54%)	26 (4.46%)		
Business and service personnel	743 (16.3%)	723 (97.31%)	20 (2.69%)		
Staff and related personnel	876 (19.2%)	838 (95.66%)	38 (4.34%)		
Professional technical personnel	1713 (37.5%)	1645 (96.03%)	68 (3.97%)		
The number of pregnancies^*^				61.130	0.000
1 pregnancy	2329 (51.0%)	2279 (97.85%)	50 (2.15%)		
2 pregnancies	1445 (31.7%)	1371 (94.88%)	74 (5.12%)		
3 or more pregnancies	790 (17.3%)	727 (92.03%)	63 (7.97%)		
Parity^*^				52.008	0.000
0 births	3146 (68.9%)	3061 (97.30%)	85 (2.70%)		
1 or more births	1418 (31.1%)	1316 (92.81%)	102 (7.19%)		
Number of spontaneous abortion				0.049	0.824
0 times	4274 (93.6%)	4099 (95.91%)	175 (4.09%)		
1 or more times	290 (6.4%)	278 (95.86%)	12 (4.14%)		
Number of drug-induced abortion^*^				23.291	0.000
0 times	3387 (74.2%)	3276 (96.72%)	111 (3.28%)		
1 or more times	1177 (25.8%)	1101 (93.54%)	76 (6.46%)		
Family history				1.956	0.376
No	4043 (88.6%)	3873 (95.80%)	170 (4.20%)		
Yes	521 (11.4%)	504 (96.74%)	17 (3.26%)		
Prepregnancy weight^*^				5.293	0.021
<56 kg	2199 (48.2%)	2123 (96.54%)	76 (3.46%)		
≥56 kg	2365 (51.8%)	2254 (95.31%)	111 (4.69%)		
BMI index^*^				12.681	0.000
<21.30 kg/m^2^	2282 (50.0%)	2211 (96.89%)	71 (3.11%)		
≥21.30 kg/m^2^	2282 (50.0%)	2166 (94.92%)	116 (5.08%)		

*P<0.05: Correlation analysis of the variables “with depressive symptoms” and other variables.

### 3.3 Multivariate logistic regression analysis

The six statistically significant independent variables identified through univariate analysis were incorporated into the multivariate logistic regression model, with the presence of depressive symptoms serving as the dependent variable. This analysis revealed that variables including the number of pregnancies, parity, an occupation in the commercial service industry, and BMI were all significantly associated with depressive symptoms, each yielding p values less than 0.05, thereby indicating statistical significance.

The analysis revealed that both increased parity (the number of times a woman has given birth) and a greater number of pregnancies were associated with an increased risk of depressive symptoms, with odds ratios (ORs) indicating that pregnant individuals with a greater number of pregnancies (OR=1.597; 95% CI: 1.249–2.042) and those with multiple births (OR=1.611; 95% CI: 1.090–2.381) were at increased risk. Similarly, a higher BMI was found to be a risk factor for prenatal depression (OR=1.476; 95% CI: 1.088-2.002), suggesting that women with higher prepregnancy BMIs are more likely to experience depressive symptoms.

Conversely, working in the business and service industries was identified as a protective factor against prenatal depression, with an OR value of less than 1 (OR = 0.610; 95% CI: 0.380–0.979). This finding indicates that women working in these sectors are at a lower risk of developing depressive symptoms during pregnancy than those with other occupation types are.

These findings underscore the importance of considering multiple demographic and health-related factors when assessing the risk of prenatal depression. The identification of both risk and protective factors is essential for developing targeted interventions aimed at mitigating the impact of prenatal depression on expectant mothers and their offspring (**Table 2**).

**Table 2:** Logistic analysis of factors influencing depressive symptoms.

Variables	β	S.E.	Wald	P	OR (95 CI)
B	-4.7615	0.3228	217.6211	<.0001	
The number of pregnancies	0.4682	0.1255	13.9170	0.0002	1.597 (1.249, 2.042)
Parity	0.4769	0.1994	5.7202	0.0168	1.611 (1.090, 2.381)
Job3	-0.4941	0.2415	4.1860	0.0408	0.610 (0.380, 0.979)
BMI index	0.3892	0.1556	6.2590	0.0124	1.476 (1.088, 2.002)
Number of medical abortion and induced abortion	-0.5821	0.3847	2.1233	0.0635	0.498 (0.129, 0.747)
Prepregnancy weight	-0.6021	0.3895	2.0192	0.0641	0.419 (0.102, 0.718)

Job3: Business and service personnel.

## 4 Discussion

### 4.1 Screening for prenatal depression symptoms

This cross-sectional survey aimed to investigate the prevalence and factors associated with prenatal depression. The study revealed that the prevalence of depression among the included prenatal individuals was 4.1%. Compared with the findings of Nilam Shakeel and others, who reported that the crude prevalence rates of prenatal depression were 13.0% globally, 8.6% in Western Europe, 19.5% in the Middle East, 17.5% in South Asia, and 11.3% in other ethnic minorities, including Eastern Europe, East Asia, South America, and Central America (18), the prevalence rate in this study was notably lower. This discrepancy could be attributed to several factors. First, the different thresholds used for the EPDS might influence the results; Shakeel et al. used an EPDS score ≥10 points as the criterion for depressive symptoms, which correlated with a sensitivity of 100% and a specificity of 87% for detecting postpartum depression in a Norwegian cohort (31). For major depressive disorder, the sensitivity and specificity of the EPDS score are 95% and 93%, respectively (32). As the EPDS score increases, so does the severity of depressive symptoms, indicating a greater risk of depression. Second, the demographic makeup of Beijing, which is predominantly Han Chinese with fewer ethnic minorities and foreigners, could lead to lower prevalence rates because less ethnic and regional variability could impact the results. Furthermore, cultural attitudes toward mental health in China, where there is a conservative approach to discussing and evaluating mental health, could lead to underreporting (33). Many prenatal individuals, particularly those who are employed, might withhold accurate descriptions of their symptoms due to fear of the negative repercussions associated with a prenatal depression diagnosis, such as workplace discrimination or social stigma. These cultural differences might contribute to a lower reported prevalence of depression among Asian populations than the international average (34).

### 4.2 Variables associated with probable depression and major depression during pregnancy

This study indicated that parity, or the number of pregnancies and births a woman has undergone, is a significant predictor of depressive symptoms during pregnancy. These findings align with those of previous studies that also identified having multiple pregnancies as a risk factor for depression during pregnancy (35–39). A study from South Africa further supported this finding, noting that pregnant individuals exhibiting depressive symptoms often have larger families (40). The underlying reasons may include the increased physical and emotional burdens faced by women, who must care for a greater number of family members. During pregnancy, the levels of estrogen and progesterone increase dramatically. Estrogen and progesterone influence mood by affecting neurotransmitter and neurosteroid levels (41, 42). Elevated cortisol levels can disrupt mood regulation and contribute to prenatal depression (43). Rapid increases in hCG levels are associated with nausea and mood changes, potentially leading to prenatal depression (44). Pregnancy induces increased secretion of adrenal cortical hormones, which can exacerbate feelings of irritability, vulnerability, and anxiety (45, 46). Additionally, elevated levels of estrogen and progesterone during pregnancy can further exacerbate depression and anxiety.

Research by Khouj MA et al. underscored a significant association between maternal depression and the number of births, with higher depression scores observed among multiparas (47). In Beijing, China, many women continue to work throughout their pregnancy, and the typical maternity leave spans approximately six months. The dual demands of maintaining work efficiency and family care, coupled with emotional and educational responsibilities toward their children, intensify as the number of children increases. Concerns about providing adequate care for existing children while managing new pregnancies can lead to stress, particularly for women with previous negative childbirth experiences, such as stillbirths, which significantly increases the risk of prenatal depression (by 3.97 times) (48).

Worries about pregnancy complications and the childbirth process further contribute to the psychological strain on expectant mothers. Studies also suggest that women with multiple children often receive less support during pregnancy and the postpartum period than first-time mothers do (38). Despite these findings, a contrasting study from Vietnam reported no significant correlation between parity and depression, highlighting potential variations influenced by regional, economic, and cultural differences (49). These discrepancies underscore the need for further research to elucidate the diverse factors contributing to prenatal depression across different settings.

This study identified a high BMI during pregnancy as a risk factor for depressive disorders, underscoring that while adequate gestational weight gain (GWG) is essential for fetal health, excessive GWG is linked to negative maternal and infant health outcomes (50–54). Evidence indicates that pregnant individuals who exceed the weight gain recommendations set by the Institute of Medicine (IOM) face significantly heightened risks of gestational diabetes, preeclampsia, and postpartum weight retention (51, 52, 54). A meta-analysis by Goldstein RF et al. further highlighted that excessive GWG increases the likelihood of cesarean delivery by 30% and the incidence of macrosomia by 95% (50). These adverse pregnancy outcomes contribute to increased delivery-related stress, potentially leading to heightened anxiety and depression among expectant mothers.

From the second trimester onward, as weight increases, pregnant individuals may become acutely aware of changes in their body shape and posture, often experiencing a decrease in mobility and a consequent reduction in participation in daily social activities, which can also impact marital relationships. The fear of not regaining one’s prepregnancy physique and the uncertainties regarding the future of one’s marriage can further exacerbate feelings of worry and insecurity (36). Although the research by Dachew BA et al. did not establish a direct correlation between excessive GWG and an increased risk of prenatal depression symptoms, maintaining an appropriate BMI during pregnancy is crucial not only for mental health but also for preventing other complications associated with excessive GWG (22, 55). This balance is vital for ensuring both physical and psychological well-being during pregnancy.

In China, occupational classifications are segmented into various categories, such as unemployed (which includes both the jobless and those out of the workforce), head of state organizations, party organizations, enterprises, institutions, business service personnel, clerks and related positions, professional and technical positions, and others. Research indicates that pregnant individuals from socioeconomically disadvantaged backgrounds exhibit the highest rates of mental disorders (56). Given the marked disparities in social and economic status in Beijing, coupled with the diverse employment statuses of pregnant individuals, it is crucial to examine how occupational types may influence pregnancy-induced depression.

Our findings reveal that pregnant individuals employed in the business and service sectors have the lowest risk of prenatal depression. Research by Bödecs T and others has demonstrated that women who are unemployed during pregnancy are more susceptible to prenatal stress and anxiety (57). Unemployment during pregnancy is prevalent and has been consistently linked to greater economic pressures, leading to family conflicts. A decline in socioeconomic status often results in reduced social support and may encourage unhealthy behaviors such as smoking and drinking (57). Additionally, isolation and feelings of inferiority stemming from a lack of social interaction, along with potential economic hardships due to decreased income, are all factors contributing to mental health disorders (57, 58).

In research conducted by González-Mesa E et al., variations in EPDS scores were observed among pregnant individuals engaged in different types of employment, with housewives, self-employed individuals, and unemployed women obtaining higher scores (59). Considering the rapid development and high demand for talent within the business and service sectors, these fields offer numerous job opportunities along with varied and enriching job content. This environment provides ample learning and development opportunities and has the advantage of stable employment. Remaining employed may foster self confidence. Given prejudices against pregnant people in the workplace, remaining employed may also be associated with harassment or maltreatment (59). This largely helps prevent family conflicts and stress, which are known independent predictors of depression in early pregnancy (58).

While temporarily setting aside the pressures and competitiveness of work, continuing employment postpregnancy may contribute to a more positive psychological state for pregnant individuals (60). Previous studies categorized job types as unemployed (housewives), teachers, doctors/engineers/lawyers, administrative workers, and business and technical workers but reported no significant correlation between job type and the incidence of prenatal depression (61). Future research could further explore the relationships among occupational type, employment status, and depression during pregnancy to enhance the understanding and development of more effective interventions.

### 4.3 Strength and limitations

This study, which had a large sample size, comprehensively explored the associations among obstetric factors, sociodemographic data, and depression among pregnant individuals through a cross-sectional analysis. It also examined the potential reasons behind the study’s findings in the context of the work and lifestyle patterns in China. This approach provides some considerations for the primary prevention of depression during pregnancy. By integrating an understanding of how various factors intersect with and influence prenatal depression, this study contributes valuable insights that could inform strategies to mitigate the risk of depression among expectant mothers in similar sociocultural settings.

This study, despite its comprehensive approach and large sample size, has several limitations that warrant consideration. First, the use of cross-sectional data inhibited the ability to establish causal relationships or to discern the temporal sequence of events. There is also the possibility that participants in the preclinical stages of depression were categorized as nondepressed, which might lead to underestimation of prevalence rates. Future studies might benefit from a longitudinal design to explore these dynamics over time. Second, the study did not incorporate more specialized psychiatric evaluations, such as the Mini-International Neuropsychiatric Interview, for pregnant individuals with high scores on the EPDS. Future research should expand the scope of the study to include such detailed psychiatric assessments following initial screenings that indicate depressive symptoms. This would help in accurately determining the actual prevalence of gestational depression in Beijing and could encompass a broader range of mental health disorders. Third, exclusion of individuals with prior mental health concerns, which may have reduced prevalence of prenatal depression. Pre pregnancy weight and pregnancy weight were self-reported *vs*. measured in a standard way. Therefore, there may be some errors in the description of weight data. Furthermore, previous research has indicated that the relationship between depressive symptoms and stages of pregnancy can vary. For example, a history of abortion may only correlate with potential depression in early pregnancy, whereas pregnancy complications may be linked specifically to severe depression in late pregnancy. Future studies should consider segmenting pregnancy into different stages and conducting more detailed investigations into how depressive symptoms may vary across these stages. This segmentation could provide deeper insights into the specific needs and preventive measures appropriate at each stage of pregnancy.

## 5 Conclusion

In summary, our findings indicate that a greater number of pregnancies and births, along with a higher BMI during pregnancy, are associated with an increased risk of depression during pregnancy. Conversely, pregnant individuals employed in commercial services have a lower risk of depression. These insights help us understand the factors influencing the development of depression during pregnancy and better predict which women are at the highest risk, enabling the timely formulation of specific intervention measures.

Additionally, the study underscores the importance of implementing comprehensive screening for mental disorders during the first trimester and integrating mental health care into routine pregnancy follow-up programs. Medical staff should proactively address the mental health concerns of pregnant individuals by providing targeted diagnosis, treatment, and health education to minimize adverse maternal and infant outcomes among high-risk groups. Moreover, social support and family care are crucial in promoting the mental health of pregnant individuals. Medical management departments are encouraged to involve family members in prenatal health education sessions, offer a variety of health education programs, and establish perinatal psychological counseling platforms to support expectant mothers throughout their pregnancies.

## Data Availability

The original contributions presented in the study are included in the article/supplementary material. Further inquiries can be directed to the corresponding authors.
